# Neogenin as a Receptor for Early Cell Fate Determination in Preimplantation Mouse Embryos

**DOI:** 10.1371/journal.pone.0101989

**Published:** 2014-07-11

**Authors:** Jae Ho Lee, Sung Sook Choi, Hae Won Kim, Wen Cheng Xiong, Churl K. Min, Sang Jin Lee

**Affiliations:** 1 Department of Nanobiomedical Sciences and WCU Research Center, Dankook University, Cheonan, S. Korea; 2 Department of Pharmacology, Sahmyook University, Seoul, S. Korea; 3 Department of Dental Biomaterials, School of Dentistry, Dankook University, Cheonan, S. Korea; 4 Institute of Molecular Medicine and Genetics, Georgia Health Science University, Augusta, Georgia, United States of America; 5 Department of Biological Sciences, Ajou University, Suwon, S. Korea; 6 Department of Animal Biotechnology, Sahmyook University, Seoul, S. Korea; National University of Singapore, Singapore

## Abstract

The first cell lineage determination in embryos takes place when two cell populations are set apart, each differentiating into the trophectoderm (TE) and inner cell mass (ICM), respectively. It is widely believed that position/polarity cues play a key role in triggering this differentiation, but it remains unclear how extracellular cues are transduced into cell fate determination. Here, we provide evidence that supports that neogenin is implicated in relaying extracellular cues into the first cell fate determination in preimplantation mouse embryos. A polarized and transient distribution of neogenin was manifested in early blastomeres. Neogenin up-regulation by its overexpression accelerated ICM development in the blastocyst concomitant with the activation of the ICM-specific transcription factors Oct3/4, Sox2, and Nanog while its depletion by small hairpin RNAs (shRNAs) caused a developmental abnormality of poorly endowed ICM accompanied by the deactivation of Oct3/4, Sox2, and Nanog. Treatment with netrin-1 among neogenin ligands further impaired both embryonic development and ICM formation while repulsive guidance molecule c (RGMc) led to opposite consequences, enhancing ICM formation. From this study, we propose a model whereby neogenin interprets its own expression level to control the first cell fate determination in response to extracellular cues.

## Introduction

Starting from fertilization and ending with implantation, preimplantation embryo development can be divided into several distinct stages: fertilization, cell cleavage, morula and blastocyst. At the early morula stage, blastomeres increase the cell-cell contact to form the compaction wherein blastomeres becomes flattened and polarized. The establishment of polarity and positional allocation among blastomeres in the compaction and at subsequent stages (late morula stage) is a major determinant in initiating cell lineage differentiation into the trophectoderm (TE) and inner cell mass (ICM) in the blastocyst [1], [2].

Two hypothetic models have been currently proposed to account for the mechanism of cell fate elaboration of the TE and ICM: The position model [3], [4] and the cell polarity model [5]. The former proposes cell lineage determination is established largely by the position of blastomeres in the late morula, a stage at which inside and outside cells become distinct. Position can be translated into cell fate. The latter proposes cell fate is established at the 8-cell stage by the establishment of cell polarity along the radius of the early morula. Subsequent cell divisions lead to symmetric or asymmetric distribution of polarity information, based on the angle of cell division. Various lines of experimental evidence have supported the two models, ranging from cell division plane-dependent inheritance of polarity information to molecular components acting as positional cues [6]–[9].

With an attempt to find a molecule that might be involved in generating and/or transmitting polarizing and positional signals in preimplantation mouse embryos, we made an interesting finding that neogenin, a member of cell-surface receptor proteins of the immunoglobulin (Ig) superfamily [10], revealed a polarized expression pattern within a single blastomere at the 8-cell stage. Neogenin was first identified as a close relative of the axon guidance receptor Deleted in Colorectal Cancer (DCC) in developing neural tissues, suggesting that it has a role in neural development [11]. Recent studies, however, have expanded this early speculation by demonstrating that neogenin has an indispensable role in many cellular functions like morphogenesis, cell migration, differentiation and apoptosis [12]. Here, we present evidence that lends support to a novel role of neogenin in developing mouse embryos, particularly, in sensing and relaying positional and/or polarity cues that are needed to lead to lineage differentiation into the TE and ICM.

## Materials and Methods

### Preparation of mouse embryos

Inbred black mouse strain C57BL mice were purchased from Oriental Bio (Sungnam, S. Korea) and maintained under a standard condition at 22±3°C, ∼60% humidity and 12-hr light/dark cycle. All procedures for animal breeding and treatment were approved by Institutional Animal Care and Use Committee of the Sahmyook University, S. Korea and conducted in accordance with the Institutional Review Board regulation of the Sahmyook University. Superovulation was induced from a 3∼5 week-old female mouse by an intraperitoneal injection of 5 IU pregnant mare serum gonadotropin (PMSG) (LG Life Sciences, Daejon, S. Korea), followed by an injection of 5 IU human chorionic gonadotropin (hCG) (LG Life Sciences) 48 hrs apart. Immediately following the hCG injection, female mice were mated with sexually mature males of the same strain. Successful mating was confirmed by the presence of a virginal plug on the female genital tract. About 18–20 hrs after the hCG injection, two-PN stage (2-PN) zygotes were retrieved from the female mouse oviduct by combining a physical removal of cumulus cells with a glass pipette and an enzymatic digestion with hyaluronidase at 300 mg/ml for 5 min at 37°C. The 2-PN zygotes were incubated in M16 media (Gibco BRL, Grand Island, NY) supplemented with 10% bovine serum albumin (BSA) at 37°C in 5% CO_2_ in a humidified chamber until a microinjection was made.

### Microinjection of neogenin cDNAs and small hairpin RNAs

The expression level of neogenin in preimplantation mouse embryos was enhanced by an injection of a vector that harbors mouse neogenin-flag fusion construct. The expression level of neogenin was knock-downed by injecting a vector that harbors a small hairpin RNA targeting neogenin. All plasmids were prepared and amplified with permissive bacteria, and the final concentrations were adjusted at 1.0 µg/ml. The 2-PN zygotes recovered from the oviduct and cultured in a micro drop of M16 media supplemented with 10% BSA under mineral oil were then mechanically denuded right before a microinjection was made. About 10 pl of each plasmid at a concentration of 1.0 µg/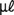 was microinjected into a zygote using a micro injector (IM-9A, Narishige, Tokyo, Japan) attached on a motorized micromanipulator (EMM-3NV, Narishige). Injection pipettes and holding pipettes were prepared from glass capillaries (BF120-94-10, Sutter, Novato, CA) by using a glass pipette puller (PC-10, Narishige). Holding pipettes were further fabricated into inner diameter of 20 µm and outer diameter of 50 µm. Injection pipettes were fabricated such that the tip length was <500 µm, and the inner diameter was 1∼2 µm. After the microinjection, the plasmid-injected 2-PN zygotes were cultured in Ham's F10 media (Gibco BRL) and observed under a fluorescence microscope (TS100, Nikon, Tokyo, Japan). All plasmid constructs used in the present studies were kindly provided by Dr. Wen Cheng Xiong at Georgia Health Sciences University, Augusta, GA.

### Immunostaining

Preimplantation mouse embryos were fixed with 4% paraformaldehyde in phosphate-buffered saline (PBS) for 30 min at 4°C, followed by a washing in PBS containing 1% BSA (PBS-BSA) three times. The embryos were then permeabilized by a 5-min incubation in PBS containing 0.1% Tween 20 at room temperature. After blocking for 30 min at room temperature in 10% normal non-immune rabbit or goat serum in PBS, the embryos were incubated with one of the following primary antibodies at 1∶100 dilution: rabbit anti-neogenin antibody (Santa Cruz Biotech, Santa Cruz, CA), goat anti-DCC antibody (Santa Cruz Biotech), rabbit anti-FAK antibody (Santa Cruz Biotech), and rabbit anti-integrin β1 antibody (Santa Cruz Biotech) in 1% PBS–BSA overnight at 4°C. After three washes with PBS, the embryos were incubated with either Alexa 488-conjugated goat secondary IgG or Alexa 568-conjugated goat secondary IgG (Invitrogen, Grand Island, NY) at 1∶500 dilution overnight at 4°C. Visualizing actin filaments were made by incubating embryos in 1 µg/ml phalloidin for 1 hr at room temperature. The nuclei were stained with DAPI at 1 µg/ml for 5 min. After three more washes with PBS, the embryos were transferred into a PBS drop on a glass slide. Fluorescent images of the stained embryos were acquired with LSM700 META confocal microscope (Carl Zeiss AG, Oberkochen, Germany).

### Reverse transcription polymerase chain reaction

Total RNAs were isolated from individual embryos by an aspiration into an injection pipette under negative pressure. About 10∼20 embryos per stage were pooled in TRIZOL reagents (Ambion, Madison, WI), and approximately 5 µg total RNA was reverse-transcribed by Superscript II reverse transcriptase in VILO RT mix (Ambion) following the manufacturer's instruction. The primers used to amplify neogenin, Sox2, Oct3/4, Nanog, Cdx2, Tead4, and β-actin were listed in Table S1. The primers were custom-designed, synthesized, and purified (Bioneer, Daejon, S. Korea). The amplification was conducted in 14 cycles of 95°C for 15 sec, 60°C for 4 min, and 58∼60°C for 4 min for cDNA pre-amplification, followed by a 10-min incubation at 95°C, after which the reaction was further subjected to 40 cycles of 95°C for 15 sec and 58∼60°C for 10 min.

### Preparation of netrin-1-enriched conditioned media

Cos-7 cells were transfected by an electroporation with the chicken netrin-1-harboring IRES EGFP plasmid. After 16 hrs in culture in normal DMEM/FBS media (Gibco BRL), netrin-1 expression was confirmed by virtue of GFP expression under a fluorescence microscope. One day after, the culture media were replaced by a culture media supplemented with a low concentration of FBS (0.5%). Conditioned media were collected starting 48 hrs after transfection, and the media were further subjected to filtration to enrich netrin-1. Enrichment for netrin-1 was verified by Western blotting with antibodies against netrin-1 (Santa Cruz Biotech). Netrin-1 conditioned culture media was collected and in storage at −80°C until use.

### Statistical analysis

All values were expressed as means ± SEM. Statistical analysis for the values was carried out using Student's *t*-test with the significance level set at P<0.05.

## Results

**Figure 1:**
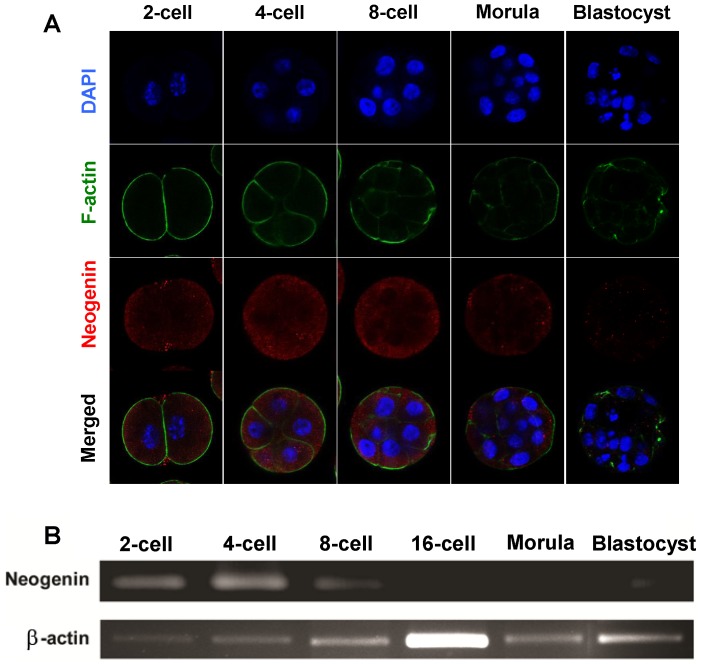
Expression of neogenin during preimplantation mouse embryo development. (A) Confocal microscopic images of neogenin expression in preimplantation mouse embryos at different developmental stages. DAPI, DAPI nuclear staining in blue; F-actin, F-actin staining in green; Neogenin, neogenin staining in red; Merged, superimposed images of DAPI, F-actin, and neogenin staining. (B) RT-PCR analysis of neogenin mRNA in preimplantation mouse embryos at different developmental stages. β-actin was used as an internal control.

Searching for a possible cellular receptor for polarity and/or positional cues implicated in early embryogenesis with a panel of antibodies, each of which recognizes an early signaling molecule involved in neurogenesis and neural path-finding, we fortuitously observed that neogenin, a member of cell-surface receptor proteins of the immunoglobulin superfamily [10], exhibited a polarized expression pattern within a single blastomere, raising an intriguing possibility that neogenin might play a role in early cell fate determination relaying extracellular stimuli. As a first step toward exploring this possibility, we examined neogenin expression pattern at various stages of preimplantation mouse embryo development by immunostaining with polyclonal anti-neogenin antibodies. Unlike its close relative Deleted in Colorectal Cancer (DCC) [11] (Fig. S1A in File S1), neogenin expression was transient, appearing as early as at the 2-cell stage and lasting until the early morula but became deficient at the late morula and blastocyst (Fig. 1A). In addition, the spatial distribution of neogenin was restricted mainly to outside cells of an embryo. A reverse transcription polymerase chain reaction (RT-PCR) analysis was consistent with early and transient nature of neogenin expression, peaking at the 4-cell stage but completely lacking at the 16-cell stage or later (Fig. 1B). By contrast, F-actin, focal adhesion kinase (FAK), or integrin did not reveal such a transient and polarized expression pattern (Fig.1A and Fig. S1B and C in File S1) as previously reported [13], [14].

**Figure 2:**
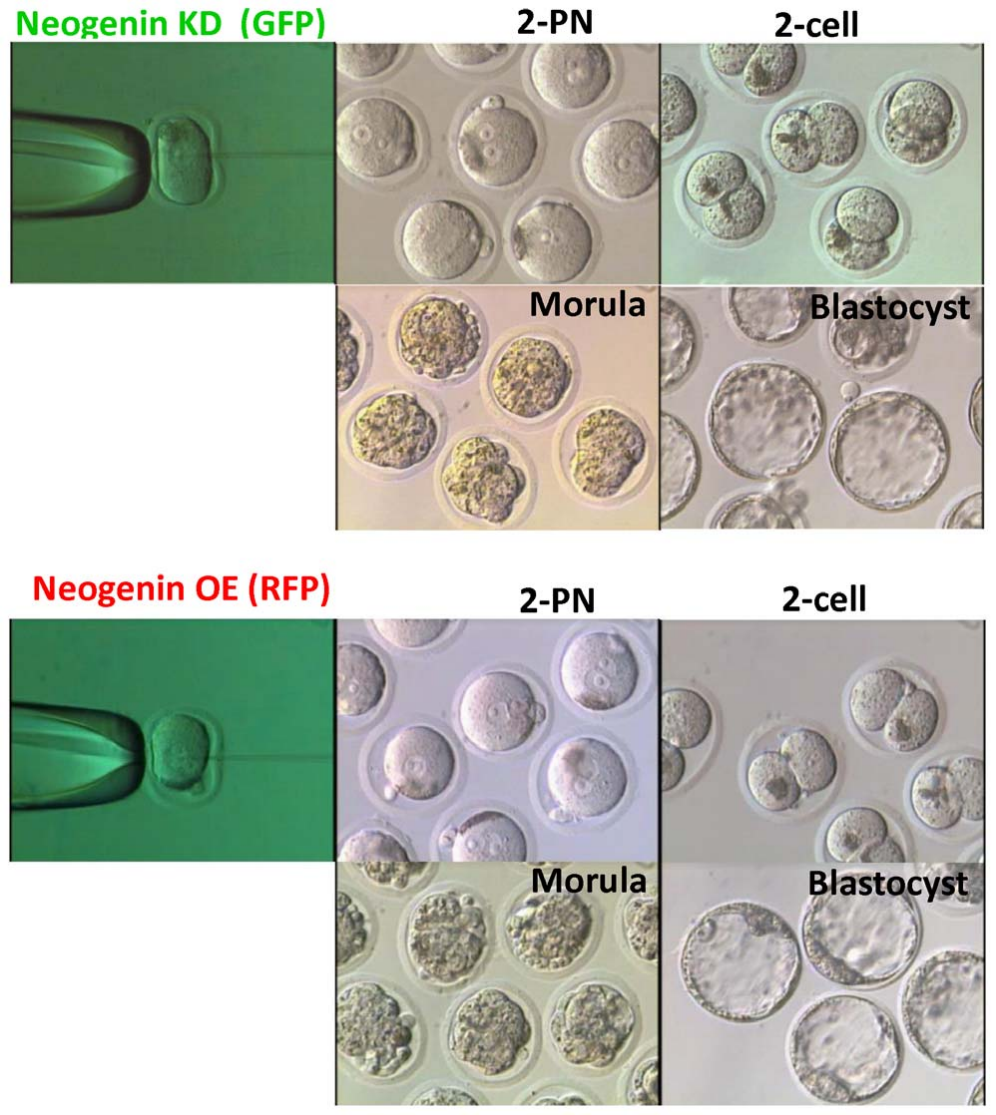
Effects of up-regulation and down-regulation of neogenin on embryo development. 2-PN mouse zygotes were microinjected with shRNA targeting neogenin (neogenin KD) or with neogenin cDNA vectors (neogenin OE) and were cultured to the blastocyst stage. To visually differentiate neogenin KD from neogenin OE embryos, GFP and RFP were co-expressed, respectively. Phase-contrast images of embryos at different developmental stages were seen.

**Figure 3:**
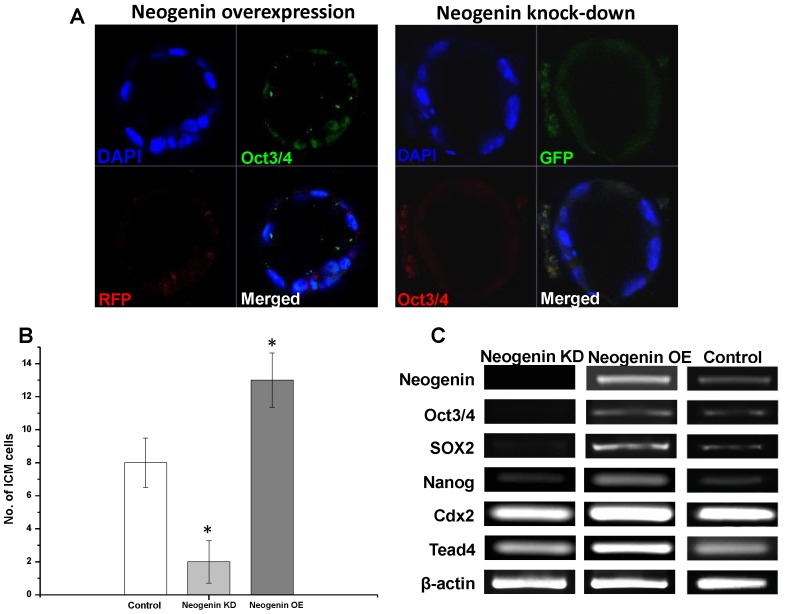
Neogenin overexpression favors ICM differentiation. (A) ICM cells in a blastocyst were viewed by immunostaining for Oct3/4 in neogenin KD and neogenin OE embryos. DAPI was for nucleus staining. (B) The number of Oct3/4-positive ICM cells in a blastocyst of control, neogenin KD, and neogenin OE embryos was represented as the mean ± SEM from three independent experiments. *Significant differences from control at *P*<0.05. (C) RT-PCR analysis of Oct3/4, Sox2, Nanog, Cdx2, and Tead4 mRNAs from blastocysts of neogenin KD, neogenin OE, and control embryos. β-actin was used as an internal control.

We next investigated whether and how neogenin was implicated in early embryonic development by microinjecting either neogenin expression vectors (neogenin overexpression, OE) or vectors harboring shRNAs targeting neogenin (neogenin knock-down, KD) into the 2-PN zygote whereby neogenin expression level was altered. To visually differentiate neogenin overexpression from neogenin knock-down, red fluorescence protein (RFP) and green fluorescence protein (GFP) were co-expressed in pairs, respectively, as an indicator (Fig. S2 in File S1), and the resulting neogenin expression level was confirmed both by immunofluorescence and by immunoblotting (Fig. S3A-C in File S1). Fig. 2 revealed representative images of embryos at each stage after receiving either neogenin shRNAs or neogenin cDNAs. There seemed to be no apparent gloss morphological difference between neogenin KD and neogenin OE embryos as well as no developmental potential differences at least until the blastocyst stage (Table 1). In fact, both the number of surviving embryos at each stage and the number of arrested embryos were not significantly different (Fig. S4A and B in File S1). To our surprise, however, ICM development was less pronounced in neogenin KD than neogenin OE embryos as evidenced by well- developed neogenin-expressing ICM cells (Fig. S3C in File S1). This finding was consistently corroborated by a finding of higher expression of ICM-specific Oct3/4 in neogenin overexpression (Fig. 3A) and by higher numbers of Oct3/4-positive ICM cells per blastocyst in neogenin OE compared to neogenin KD embryos (Fig. 3B), strongly suggesting that neogenin might be involved in the first cell lineage determination. To further test this possibility, we carried out a series of experiments to reveal whether the first cell fate specification is linked to the signal cascade downstream of neogenin. First of all, we examined whether the expression of Oct3/4, Sox2, and Nanog, key transcriptional regulators for ICM differentiation/maintenance [15]–[18], was influenced by neogenin expression level. RT-PCR analyses showed a strong correlation existed between expression of three transcription factors and that of neogenin. In neogenin KD blastocysts, none of Oct3/4, Sox2, and Nanog was detected, which sharply contrasted to a significant increase in expression of all three transcription factors upon neogenin overexpression compared to control embryos (Fig. 3C). Unexpectedly, the expression level of Cdx2 and Tead4, transcription factors implicated in TE differentiation/maintenance [19]–[22], was less influenced by expression level of neogenin, validating that up-regulation of neogenin leads to activation of transcriptional regulators specific for the ICM but not for the TE establishment.

**Table 1:** Developmental potentials of 2-PN mouse zygotes after receiving neogenin shRNAs or neogenin cDNAs.

	No. of 2-PN zygotes examined	No. of 2-PN zygotes injected	No. of embryos developing to			
			2-cell	4∼8-cell	Morula	Blastocyst
Control	90	90	87 (96.7%)	87 (96.7%)	83 (92.2%)	78 (86.7%)
Neogenin knock-down	218	206	201 (97.6%)	194 (94.2%)	185 (89.8%)	172 (83.5%)
Neogenin overexpression	218	190	184 (96.8%)	178 (93.7%)	167 (87.9%)	159 (83.7%)

**Figure 4:**
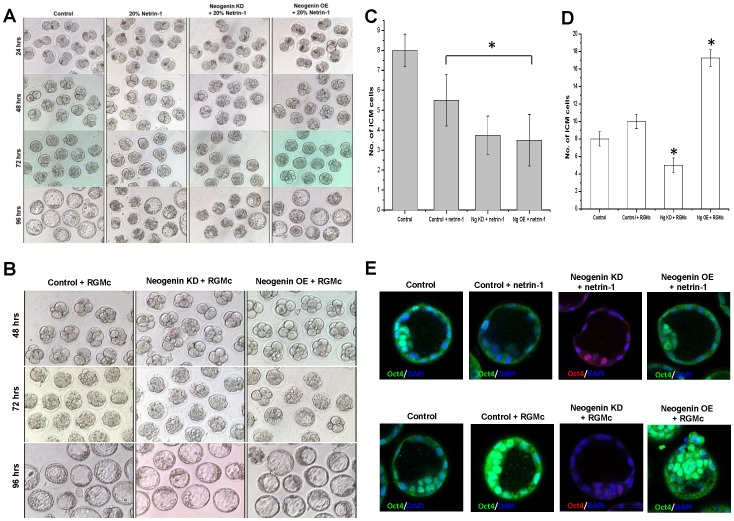
Neogenin ligands netrin-1 and RGMc exert an antagonistic effect on ICM differentiation. (A) Netrin-1-enriched conditioned media was added to the culture media at 20% (v/v), and the gross morphology of developing mouse embryos was observed over 96 hr-period. Control, normal embryos without netrin-1; 20% Netrin-1, normal embryos in 20% netrin-1; Neogenin KD+20% netrin-1, neogenin knock-down embryos in 20% netrin-1; Neogenin OE+20% netrin-1, neogenin overexpressing embryos in 20% netrin-1. (B) Recombinant RGMc was added to culture media at 10 ng/ml and the gross morphology of developing mouse embryos over 96 hrs in culture was observed. Control + RGMc, control embryos in culture media supplemented with recombinant RGMc; Neogenin KD + RGMc, neogenin knock-down embryos in culture media supplemented with recombinant RGMc; Neogenin OE + RGMc, neogenin overexpressing embryos in culture media supplemented with recombinant RGMc. (C) ICM cells in a blastocyst was counted from different treatments: Control, no treatment on normal embryos; Control + netrin-1, 20% netrin-1 treatment on normal embryos; Ng KD + netrin-1, 20% netrin-1 treatment on neogenin knock-down embryos; Ng OE + netrin-1, 20% netrin-1 treatment on neogenin overexpressing embryos. (D) ICM cells in a blastocyst was counted from different treatments: Control, no treatment on normal embryos; Control + RGMc, 10 ng/ml RGMc treatment on normal embryos; Ng KD + RGMc, 10 ng/ml RGMc treatment on neogenin knock-down embryos; Ng OE + RGMc, 10 ng/ml RGMc treatment on neogenin overexpressing embryos (E) Immunofluorescent staining for Oct3/4 after receiving different combinations of experimental treatment as described in C and D. *Significant differences from control at *P*<0.05.

In view to further demonstrate effectiveness of neogenin up-regulation toward the first cell lineage specification, we next analyzed phenotypic outcomes of treatment of embryos with two neogenin ligands, netrin-1 and repulsive guidance molecule c (RGMc) [23]–[26]. Netrin-1 was made available by enriching the conditioned media in which Cos-7 cells transfected with chicken netrin-1 expression vectors were cultured for two days and its enrichment for netrin-1 was confirmed by immunoblotting (Fig. S5 in File S1). Two-PN zygotes were cultured to the blastocyst in media supplemented with either netrin-1 enriched media at 20% (v/v) or recombinant RGMc at 10 ng/ml, and their effects on the gross morphology of developing embryos were monitored. Netrin-1 treatment caused fewer embryos to reach the blastocyst, most embryos being arrested at the morula stage and its development-arresting effect waxed strong by neogenin knock-down and was partially rescued by neogenin overexpression. Furthermore, netrin-1-treated embryos had a significantly lower number of ICM cells than control (Fig. 4A, C). To the contrary, RGMc treatment caused no apparent development arrest but rather produced a converse response in embryo development. Neogenin overexpression increased the fraction of blastocysts with a higher number of ICM cells in the presence of RGMc whereas neogenin knock-down led to the establishment of the blastocysts with less pronounced ICM formation even in the presence of RGMc (Fig 4B, D and Fig. S6 and Fig. S7 in File S1). A quantitative analysis revealed that the presence of netrin-1 at 20% itself hampered normal embryo development to the blastocyst by as much as 50% (Table 2) whereas RGMc did not (Table 3). Immunostaining for Oct3/4 further confirmed our finding that a combination of RGMc treatment and neogenin overexpression yielded the highest number of ICM cells while a combination of netrin-1 treatment and neogenin knock-down yielded the fewest ICM cells in the blastocyst (Fig. 4E). Collectively, these observations led us to propose that neogenin has a dual function in the first cell lineage specification. The choice between a progression to and aversion from ICM formation is governed by the extent of neogenin expression at as late as the 8-cell stage. When neogenin is down-regulated, polarity and/or positional cues tilt toward the formation of the blastocyst with less pronounced ICM. On the other hand, when neogenin is up-regulated, environmental cues favor the formation of the blastocyst with a fully developed ICM.

**Table 2:** Developmental potential of 2-PN zygotes cultured with netrin-1.

Treatment	No. of 2-PN embryos examined	No. of embryos developing to			
		2-Cell	4∼8-Cell	Morula	Blastocyst
Normal embryo	20	19 (95.0%)	19 (95.0%)	19 (95.0%)	19 (95.0%)
Normal embryo in 20% netrin-1	20	20 (100.0%)	18 (90.0%)	18 (90.0%)	9 (45.0%)
Neogenin KD embryo in 20% netrin-1	23	22 (95.7%)	19 (82.6%)	22 (95.7%)	10 (43.5%)
Neogenin OE embryo in 20% netrin-1	30	29 (96.7%)	28 (93.3%)	27 (90.0%)	17 (56.7%)

**Table 3:** Development potential of 2-PN zygotes cultured with RGMc.

Treatment	No. of 2-PN embryos examined	No. of embryos developing to			
		2-cell	4-8-cell	Morula	Blastocyst
Normal embryo	28	28 (100.0%)	28 (100.0%)	27 (96.4%)	22 (78.5%)
Normal embryo in 10 ng/ml RGMc	30	30 (100.0%)	24 (80.0%)	27 (90.0%)	23 (76.7%)
Neogenin KD embryo in 10 ng/ml RGMc	72	70 (97.2%)	57 (79.2%)	62 (86.1%)	45 (62.5%)
Neogenin OE embryo in 10 ng/ml RGMc	65	64 (98.5%)	56 (86.2%)	53 (81.5%)	41 (63.1%)

## Discussion

Our results provide what is to our knowledge the first evidence that neogenin may act as a receptor for extracellular cues in the first cell fate determination in preimplantation mouse embryos. Supporting evidence includes (*i*) a polarized and stage-specific expression of neogenin during early embryo development, (*ii*) alteration of neogenin expression, down-regulation or up-regulation, yields differential consequences to embryo development, (*iii*) neogenin ligand RGMc favors ICM formation but to varying extents depending upon the expression level of neogenin in such a way that at high neogenin expression, RGMc promotes differentiation of early blastomeres into thus an outgrowth of the ICM, whereas at its low expression, netrin-1 antagonizes differentiation of early blastomeres into the ICM, and (*iv*) up-regulation of neogenin triggers activation of Oct3/4, Sox2, and Nanog, transcriptional regulators, whose expression signify cellular undifferentiation and pluripotency, accompanied by early blastomere differentiation into the ICM while neogenin down-regulation abrogates their activation.

Since neogenin was identified as a receptor for the neuronal axon guidance cues netrins and RGMs, it has been implicated in such diverse developmental processes as neuronal development, mammary gland development, endochondrial bone formation, and cell cycle progression [10], [27]–[29]. One of the common themes emerging from these studies was that the neogenin signaling may provide a potential convergent point for cross talks among different extracellular cues, chemical or physical in nature, by which cellular differentiation and development are exactly executed to maintain developmental integrity in a permissive milieu. Despite a lack of supporting evidence, this line of view tempted us to raises an intriguing possibility that the establishment of cell polarity may result from a signaling initiated from neogenin that receives temporal and spatial input from various extracellular stimuli. It also suggests that the neogenin signaling pathway is, therefore, richly endowed with versatility needed to allow development- and growth-triggering signal entry to effectively couple specific extracellular stimuli to appropriate cellular responses. Based on our studies and others [27], [30], the neogenin signaling seems a perfect fit for this role. In other words, neogenin activation by extracellular stimuli may well act as a cellular switch to turn on and off a signaling that is indispensable for early blastomere commitment to their cell fate specification. Although neogenin is known to be a multifunctional receptor, yet signaling pathway activated by neogenin are poorly understood, this notion is nicely illustrated by a recent finding showing that neogenin facilitates BMP/Smad signaling by providing the physical link between neogenin and the BMP receptor [27]. Our studies also demonstrated neogenin implication in activation of Oct3/4, Sox2, and Nanog, signaling molecules playing a key role in maintenance of undifferentiation and pluripotency of a cell.

One of the key findings in our studies was that the ICM cell number in a blastocyst was markedly increased by elevated neogenin expression and its ligand RGMc in a synergistic manner. Then, next intriguing questions would be whether this phenotypic change is an outcome of increased internalization of cells at the transition from the 8-cell stage to the 64-cell stage or results from enhanced proliferation of pre-existing inside cells. Currently, our working hypothesis favors enhanced proliferation of inside cells after the internalization is completed by the asymmetrical cell division, which commences at the 8-cell stage. Supporting our reasoning, it has been recently proposed that cell repositioning due to their movement among the ICM cells is prerequisite for EPI and PE lineage specification while a wrong positioning was liable to apoptotic cell death [31]. We hypothesize that after internalization, more inside cells would be tuned to survive and proliferate when their neogenin signaling is up-regulated, leading to increased numbers of ICM cells. This idea is in general accordance with Morris et al. [32] in that differential cell signaling in cells internalized at different times influences lineage segregation.

The effects of two seemingly antagonistic neogenin ligands netrin-1 and RGMc in terms of embryonic development deserve further comments. Unlike RGMc, netrin-1 caused an embryonic development arrest at the morula. Is it an artifact? Netrin-1 was made available by enriching the conditioned media of Cos-7 cells that were transfected with chicken netrin-1 expression vectors, and externally added to embryos. The fact that development-arresting effect of netin-1 was exacerbated by neogenin knock-down but partially rescued by neogenin overexpression strongly argues against this possibility. Then, what is the underlying mechanism for the netrin-1-induced developmental arrest? Based on our observation that neogenin expression alteration, whether overexpressed or knock-down, did not apparently cause an embryonic development arrest, netrin-1 may reveal the antagonism against its receptor neogenin in mouse embryos, thereby conferring a detrimental effect on embryo development. The antagonism between netrin-1 and its receptor depending on different physiological context is not an exception [33].

**Figure 5:**
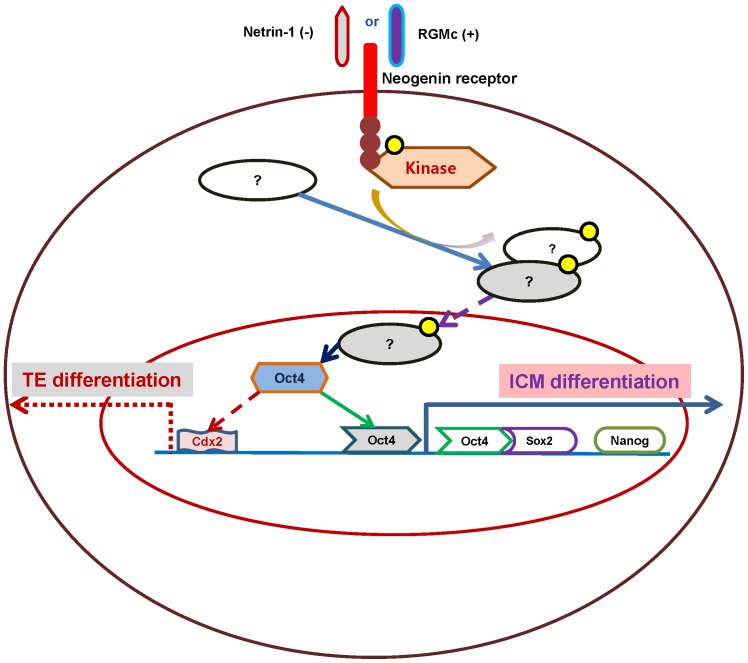
A model for downstream signaling of neogenin and regulation of early cell fate determination in preimplantation mouse embryos. The differential activation of neogenin signaling either by neogenin expression level or its ligation with ligands, which in turn receive thus relaying temporal and spatial input from various external stimuli during early embryo development leads to the establishment of the first cell fate specification.

Remaining questions are: (*i*) what are the physiological context wherein these extracellular cues are linked to regulation of cell differentiation, (*ii*) what are molecular components acting as positional/polarity cues, and (*iii*) how the cell polarity or positional cues make their way to regulators of cell lineage specification. Based on present results, the expression level of neogenin could dictate the physiological context for the first cell fate determination: a moderate level of neogenin expression in blastomeres prior to the morula stage ensures its ligation with ligands triggers and thereby maintains a balanced differentiation between the ICM and TE. However, when neogenin expression level is altered, either high or low, two ligands, RGMc and netrin-1, drive cellular differentiation in opposite direction, *i.e.* into more ICM differentiation and less ICM differentiation, respectively, by activating a signaling pathway(s) whose downstream effectors act on either Oct3/4 or Cdx2 (Fig. 5). More importantly, our findings have provided a novel framework from which a novel means to generate embryonic stem cells is made possible, *e.g*., by selectively enhancing ICM differentiation as a result of neogenin overexpression and/or its ligand treatment. These studies have also illustrated how the activity of neogenin integrates with other molecular machinery to implement and stabilize the initial asymmetry created by the localized activation of Oct3/4 and Cdx2, suggesting that integration of neogenin signaling activity with other signaling pathways may be a general feature of cellular polarization and differentiation.

## Conclusions

Our data showed a transient and polarized expression of neogenin in early blastomeres. The expression level of neogenin was closely related to cellular differentiation into the ICM in association with up- and down-regulation of signaling molecules Oct3/4, Sox2, and Nanog. We also identified two neogenin ligands, RGMc and netrin-1, antagonistically act as potential modulators of the first cell fate specification. Collectively, our data strongly supports the involvement of neogenin in the first cell fate decision by activating a signal transduction pathway whereby extracellular cues could be relayed and converted into the first cell fate determination in preimplantation mouse embryos.

## Supporting Information

Table S1 **Sequences of the primers and PCR conditions used for RT-PCR.**
(DOCX)

File S1 **Supporting Figures. Figure S1,** Expression profiles of focal adhesion kinase (FAK), F-action, integrin β1 subunit in mouse embryos viewed by immunostaining during early embryo development. Preimplantation mouse embryos at various developmental stages were subjected to immunostaining for DCC in A; integrin β1 subunit and F-actin in B; FAK and F-actin in C. DAPI was for nuclear staining. **Figure S2,** Green fluorescence protein (GFP) and red fluorescence protein (RFP) expression as an indicator of neogenin knock down and neogenin overexpression, respectively. After microinjection of neogenin-targeting shRNA vector that harbors conjugated GFP or co-injection of the neogenin cDNA vector and RFP vector into the 2-PN zygotes, the expression of GFP and RFP was visualized under a fluorescence microscope at the 2-cell and 4-cell stage, respectively. Left panel, phase-contrast images; middle and right panels, fluorescence images. **Figure S3,** Expression of neogenin in a blastocyst after microinjecting small hairpin RNA neogenin targeting vectors. (A) After microinjection of neogenin targeting shRNA vector into 2-PN zygotes, the expression level of neogenin in individual cells in a blastocyst was evaluated by immunostaining with anti-flag antibodies. In the left panel, scrambled neogenin shRNA vectors were microinjected (control). In the right panel, neogenin-targeting shRNA vectors were microinjected. DAPI, DAPI nucleus staining in blue; Anti-flag, visualization of the flag tag on neogenin in red; GFP, green fluorescence proteins in green; merged, superimposition of DAPI, anti-flag, and GFP. (B) Whole cell lysates of blastocysts were immunoblotted with anti-flag antibodies. GFP was used as a loading control. Scrambled shRNA, scrambled neogenin shRNA injection; Ng shRNA, neogenin-targeting shRNA injection. (C) The neogenin cDNA vectors or the neogenin-targeting shRNA vectors were microinjected into 2-PN zygotes and resulting blastocysts were subjected to immunostaining with anti-neogenin antibodies to reveal the expression level of neogenin. In the upper panel, neogenin-targeting shRNAs were injected. Blastocysts were immunostained with anti-neogenin antibodies in red. GFP, green fluorescence proteins in green; Merged, superimposition of DAPI, anti-neogenin, and GFP. In the lower panel, neogenin cDNA vectors and RFP vectors were co-injected. Blastocysts were immunostained with anti-neogenin antibodies in green. DAPI, DAPI nuclear staining in blue; RFP, red fluorescence protein in red; merged, superimposition of DAPI, RFP, and anti-neogenin. **Figure S4,** The developmental potential of neogenin overexpressing and neogenin knock-down embryos. (A) The number of surviving mouse embryos at each developmental stage after receiving neogenin shRNA or neogenin cDNA at 2-PN zygotes was counted. (B) The number of developmentally arrested embryos was counted after receiving the same treatment. **Figure S5,** Production of netrin-1-enriched conditioned media. Cos-7 cells were transfected with chicken netrin-1 vectors by electroporation. Two days after transfection, the culture media were collected and filtered, and subjected to immunoblotting with anti-netrin-1 antibodies. Cell lysates, netrin-1 transfected Cos-7 cell lysates; conditioned media, netrin-1-enriched conditioned media. **Figure S6,** The gross morphology of neogenin knock-down mouse blastocysts. The neogenin KD embryos were selected by virtue of green fluorescence from GFP at the 2-cell stage and further incubated in the embryo culture media supplemented with RGMc at 10 ng/ml for 96 hrs to reach the blastocyst. The green arrows indicate blastocysts with little, if any, inner cell mass. **Figure S7,** The gross morphology of neogenin-overexpressing mouse blastocysts. The neogenin OE embryos were selected by virtue of red fluorescence from RFP at the 2-cell stage and further incubated in the embryo culture media supplemented with RGMc at 10 ng/ml for 96 hrs until reaching the blastocyst. The red arrows indicate blastocysts having well-developed ICM.
(ZIP)
