# Correction: Neogenin as a Receptor for Early Cell Fate Determination in Preimplantation Mouse Embryos

**DOI:** 10.1371/journal.pone.0117866

**Published:** 2015-02-03

**Authors:** The PLOS ONE Staff

The third author’s name is spelled incorrectly. The correct name is: Hae-Won Kim. The correct citation is: Lee JH, Choi SS, Kim H-W, Xiong WC, Min CK, Lee SJ, et al. (2014) Neogenin as a Receptor for Early Cell Fate Determination in Preimplantation Mouse Embryos. PLoS ONE 9(7): e101989. doi:10.1371/journal.pone.0101989.

There is an error in affiliation 1 for authors Jae Ho Lee and Hae-Won Kim. Affiliation 1 should be: Department of Nanobiomedical Sciences and BK21 PLUS MBM Global Research Center for Regenerative Medicine, Dankook University, Cheonan, S. Korea.
