# Supplementary material for: Neogenin as a Receptor for Early Cell Fate Determination in Preimplantation Mouse Embryos
Source: PLoS One. 2014 Jul 11;9(7):e101989. doi: 10.1371/journal.pone.0101989 (PMC4094428; doi:10.1371/journal.pone.0101989)
Supplement: Table S1 — Sequences of the primers and PCR conditions used for RT-PCR. (DOCX) [file pone.0101989.s001.docx]

**Table S1. Sequences of the primers and PCR conditions used for RT-PCR**

| **Primer name** | **Forward primer**  **Backward primer** | **PCR condition** | |
| --- | --- | --- | --- |
| Neogenin | 5′-TACACTCCAGTGCCAGATCC-3′  5′-GCCGGGTACAAAAGACAGCA-3′ | | 2.5 mM MgCl  60^o^C |
| Oct3/4 | 5’-CACGAGTGGAAAGCAACTCA-3‘  5’-AGATGGTGGTCTGGCTGAAC-3’ | | 58^o^C |
| Sox2 | 5’-GGTTACCTCTTCCTCCCACTCCAG-3’  5’-TCACATGTGCGACAGGGGCAG-3’ | | 58^o^C |
| Nanog | 5’-CACCCACCCATGCTAGTCTT-3’  5’-ACCCTCAAACTCCTGGTCCT-3’ | | 60^o^C |
| Tead4 | 5’-AGCTAAGAACAAGGCCCTGC-3’  5’-TGCCAAAACCCTGAGATTGC-3’ | | 58^o^C |
| Cdx2 | 5’-GCAGTCCCTAGGAAGCCAAGTGA-3’  5’-CTCTCGGAGAGCCCAAGTGTG-3’ | | 58^o^C |
| β-actin | 5’-TGTATGCCTCTGGTCGTACCACAG-3’  5’-GATGTCACGCACGATTTCCCTCTC-3’ | | 58^o^C |
