# Supplementary figures and images for: Neogenin as a Receptor for Early Cell Fate Determination in Preimplantation Mouse Embryos
Source: PLoS One. 2014 Jul 11;9(7):e101989. doi: 10.1371/journal.pone.0101989 (PMC4094428; doi:10.1371/journal.pone.0101989)

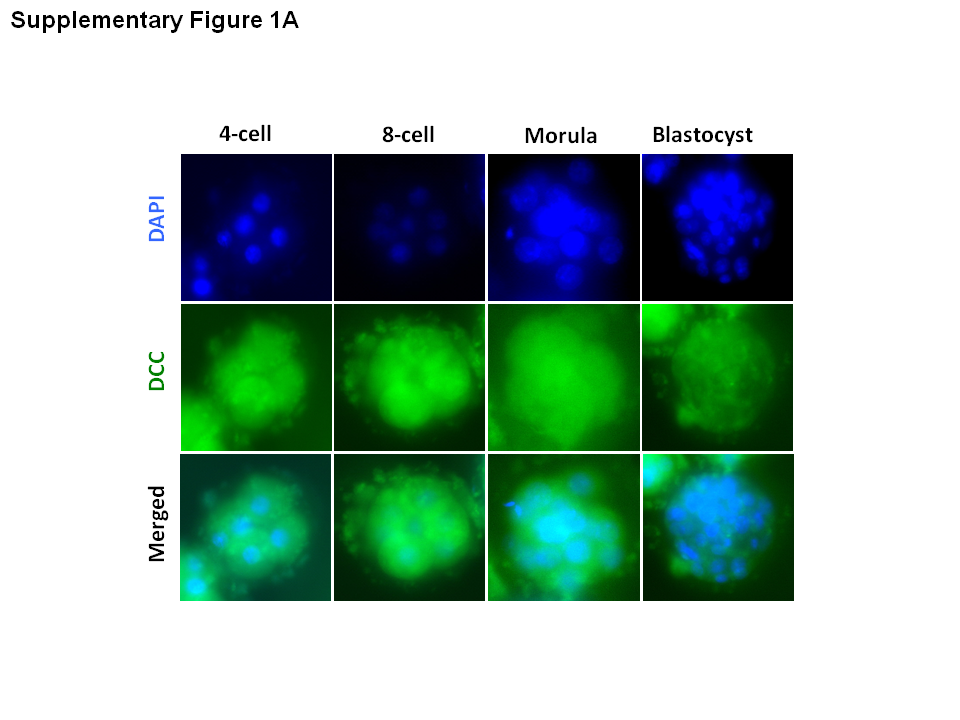

Supplement: File S1 — Supporting Figures. Figure S1, Expression profiles of focal adhesion kinase (FAK), F-action, integrin β1 subunit in mouse embryos viewed by immunostaining during early embryo development. Preimplantation mouse embryos at various developmental stages were subjected to immunostaining for DCC in A; integrin β1 subunit and F-actin in B; FAK and F-actin in C. DAPI was for nuclear staining. Figure S2, Green fluorescence protein (GFP) and red fluorescence protein (RFP) expression as an indicator of neogenin knock down and neogenin overexpression, respectively. After microinjection of neogenin-targeting shRNA vector that harbors conjugated GFP or co-injection of the neogenin cDNA vector and RFP vector into the 2-PN zygotes, the expression of GFP and RFP was visualized under a fluorescence microscope at the 2-cell and 4-cell stage, respectively. Left panel, phase-contrast images; middle and right panels, fluorescence images. Figure S3, Expression of neogenin in a blastocyst after microinjecting small hairpin RNA neogenin targeting vectors. (A) After microinjection of neogenin targeting shRNA vector into 2-PN zygotes, the expression level of neogenin in individual cells in a blastocyst was evaluated by immunostaining with anti-flag antibodies. In the left panel, scrambled neogenin shRNA vectors were microinjected (control). In the right panel, neogenin-targeting shRNA vectors were microinjected. DAPI, DAPI nucleus staining in blue; Anti-flag, visualization of the flag tag on neogenin in red; GFP, green fluorescence proteins in green; merged, superimposition of DAPI, anti-flag, and GFP. (B) Whole cell lysates of blastocysts were immunoblotted with anti-flag antibodies. GFP was used as a loading control. Scrambled shRNA, scrambled neogenin shRNA injection; Ng shRNA, neogenin-targeting shRNA injection. (C) The neogenin cDNA vectors or the neogenin-targeting shRNA vectors were microinjected into 2-PN zygotes and resulting blastocysts were subjected to immunostaining with anti-neo [file pone.0101989.s002.zip › Figure S1A.TIF]

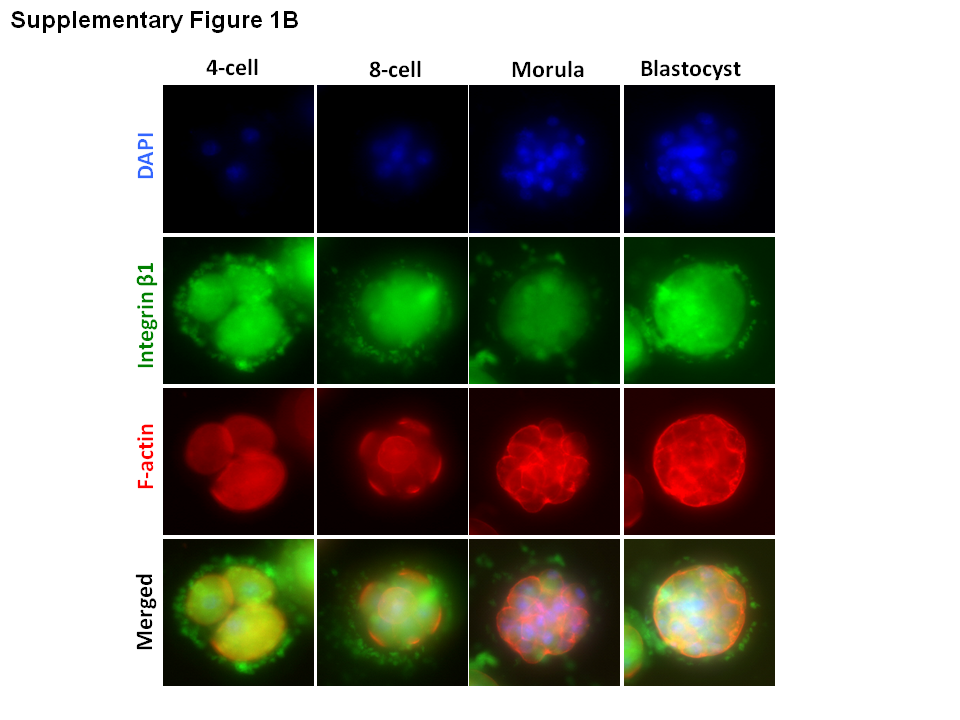

Supplement: File S1 — Supporting Figures. Figure S1, Expression profiles of focal adhesion kinase (FAK), F-action, integrin β1 subunit in mouse embryos viewed by immunostaining during early embryo development. Preimplantation mouse embryos at various developmental stages were subjected to immunostaining for DCC in A; integrin β1 subunit and F-actin in B; FAK and F-actin in C. DAPI was for nuclear staining. Figure S2, Green fluorescence protein (GFP) and red fluorescence protein (RFP) expression as an indicator of neogenin knock down and neogenin overexpression, respectively. After microinjection of neogenin-targeting shRNA vector that harbors conjugated GFP or co-injection of the neogenin cDNA vector and RFP vector into the 2-PN zygotes, the expression of GFP and RFP was visualized under a fluorescence microscope at the 2-cell and 4-cell stage, respectively. Left panel, phase-contrast images; middle and right panels, fluorescence images. Figure S3, Expression of neogenin in a blastocyst after microinjecting small hairpin RNA neogenin targeting vectors. (A) After microinjection of neogenin targeting shRNA vector into 2-PN zygotes, the expression level of neogenin in individual cells in a blastocyst was evaluated by immunostaining with anti-flag antibodies. In the left panel, scrambled neogenin shRNA vectors were microinjected (control). In the right panel, neogenin-targeting shRNA vectors were microinjected. DAPI, DAPI nucleus staining in blue; Anti-flag, visualization of the flag tag on neogenin in red; GFP, green fluorescence proteins in green; merged, superimposition of DAPI, anti-flag, and GFP. (B) Whole cell lysates of blastocysts were immunoblotted with anti-flag antibodies. GFP was used as a loading control. Scrambled shRNA, scrambled neogenin shRNA injection; Ng shRNA, neogenin-targeting shRNA injection. (C) The neogenin cDNA vectors or the neogenin-targeting shRNA vectors were microinjected into 2-PN zygotes and resulting blastocysts were subjected to immunostaining with anti-neo [file pone.0101989.s002.zip › Figure S1B.TIF]

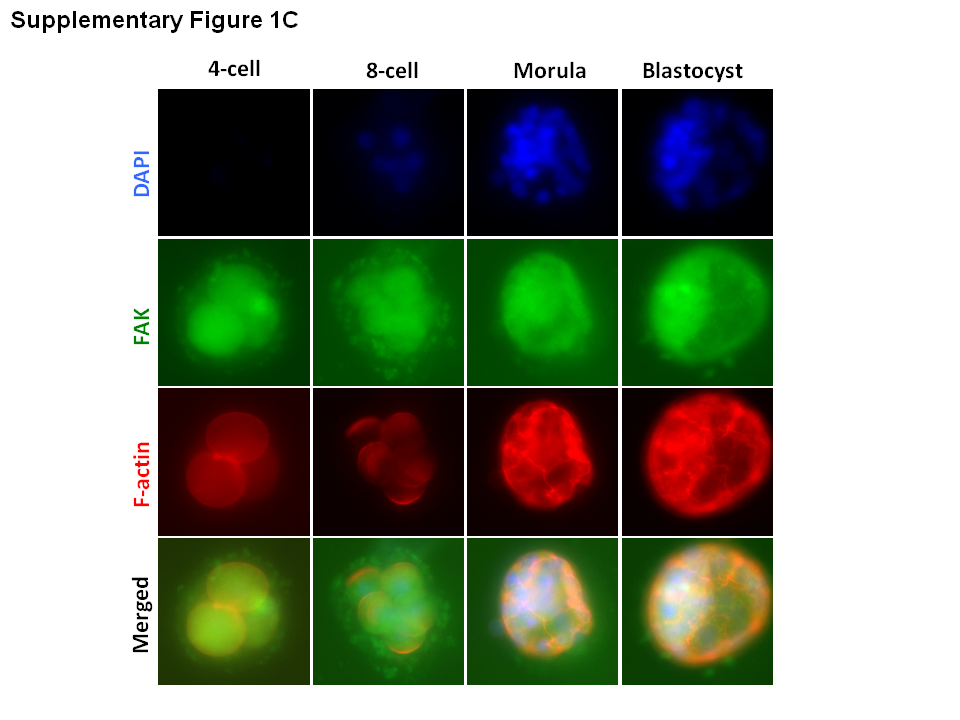

Supplement: File S1 — Supporting Figures. Figure S1, Expression profiles of focal adhesion kinase (FAK), F-action, integrin β1 subunit in mouse embryos viewed by immunostaining during early embryo development. Preimplantation mouse embryos at various developmental stages were subjected to immunostaining for DCC in A; integrin β1 subunit and F-actin in B; FAK and F-actin in C. DAPI was for nuclear staining. Figure S2, Green fluorescence protein (GFP) and red fluorescence protein (RFP) expression as an indicator of neogenin knock down and neogenin overexpression, respectively. After microinjection of neogenin-targeting shRNA vector that harbors conjugated GFP or co-injection of the neogenin cDNA vector and RFP vector into the 2-PN zygotes, the expression of GFP and RFP was visualized under a fluorescence microscope at the 2-cell and 4-cell stage, respectively. Left panel, phase-contrast images; middle and right panels, fluorescence images. Figure S3, Expression of neogenin in a blastocyst after microinjecting small hairpin RNA neogenin targeting vectors. (A) After microinjection of neogenin targeting shRNA vector into 2-PN zygotes, the expression level of neogenin in individual cells in a blastocyst was evaluated by immunostaining with anti-flag antibodies. In the left panel, scrambled neogenin shRNA vectors were microinjected (control). In the right panel, neogenin-targeting shRNA vectors were microinjected. DAPI, DAPI nucleus staining in blue; Anti-flag, visualization of the flag tag on neogenin in red; GFP, green fluorescence proteins in green; merged, superimposition of DAPI, anti-flag, and GFP. (B) Whole cell lysates of blastocysts were immunoblotted with anti-flag antibodies. GFP was used as a loading control. Scrambled shRNA, scrambled neogenin shRNA injection; Ng shRNA, neogenin-targeting shRNA injection. (C) The neogenin cDNA vectors or the neogenin-targeting shRNA vectors were microinjected into 2-PN zygotes and resulting blastocysts were subjected to immunostaining with anti-neo [file pone.0101989.s002.zip › Figure S1C.TIF]

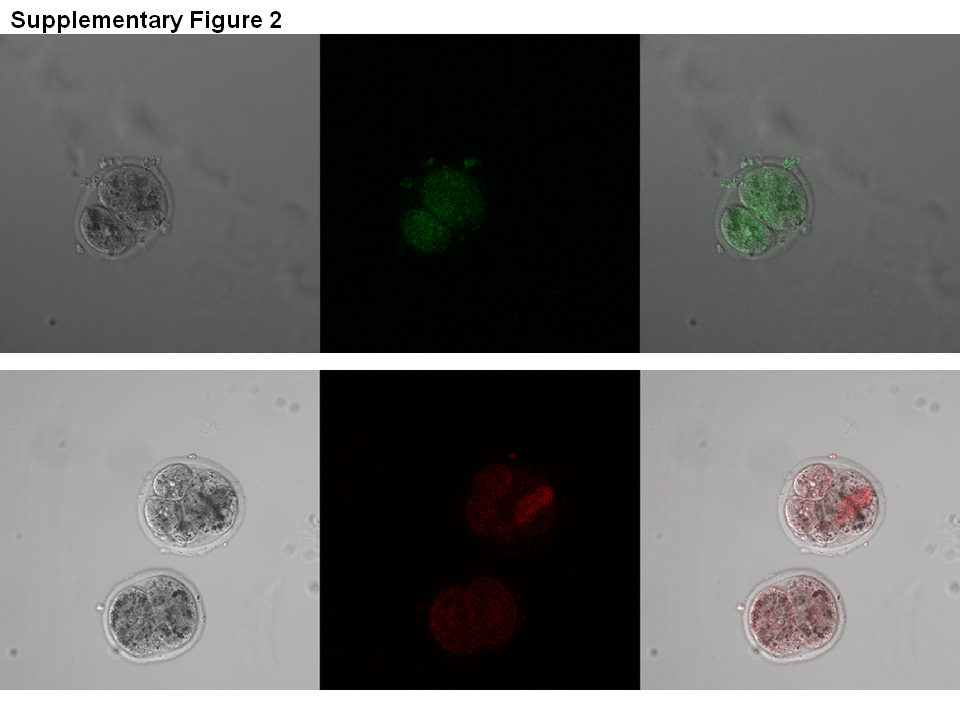

Supplement: File S1 — Supporting Figures. Figure S1, Expression profiles of focal adhesion kinase (FAK), F-action, integrin β1 subunit in mouse embryos viewed by immunostaining during early embryo development. Preimplantation mouse embryos at various developmental stages were subjected to immunostaining for DCC in A; integrin β1 subunit and F-actin in B; FAK and F-actin in C. DAPI was for nuclear staining. Figure S2, Green fluorescence protein (GFP) and red fluorescence protein (RFP) expression as an indicator of neogenin knock down and neogenin overexpression, respectively. After microinjection of neogenin-targeting shRNA vector that harbors conjugated GFP or co-injection of the neogenin cDNA vector and RFP vector into the 2-PN zygotes, the expression of GFP and RFP was visualized under a fluorescence microscope at the 2-cell and 4-cell stage, respectively. Left panel, phase-contrast images; middle and right panels, fluorescence images. Figure S3, Expression of neogenin in a blastocyst after microinjecting small hairpin RNA neogenin targeting vectors. (A) After microinjection of neogenin targeting shRNA vector into 2-PN zygotes, the expression level of neogenin in individual cells in a blastocyst was evaluated by immunostaining with anti-flag antibodies. In the left panel, scrambled neogenin shRNA vectors were microinjected (control). In the right panel, neogenin-targeting shRNA vectors were microinjected. DAPI, DAPI nucleus staining in blue; Anti-flag, visualization of the flag tag on neogenin in red; GFP, green fluorescence proteins in green; merged, superimposition of DAPI, anti-flag, and GFP. (B) Whole cell lysates of blastocysts were immunoblotted with anti-flag antibodies. GFP was used as a loading control. Scrambled shRNA, scrambled neogenin shRNA injection; Ng shRNA, neogenin-targeting shRNA injection. (C) The neogenin cDNA vectors or the neogenin-targeting shRNA vectors were microinjected into 2-PN zygotes and resulting blastocysts were subjected to immunostaining with anti-neo [file pone.0101989.s002.zip › Figure S2.TIF]

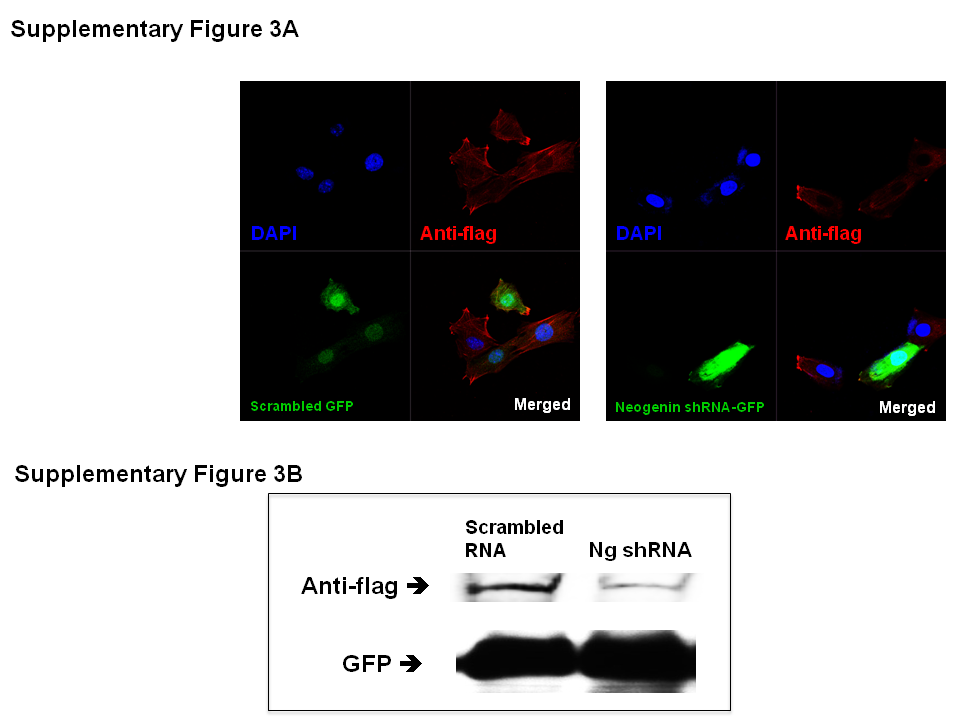

Supplement: File S1 — Supporting Figures. Figure S1, Expression profiles of focal adhesion kinase (FAK), F-action, integrin β1 subunit in mouse embryos viewed by immunostaining during early embryo development. Preimplantation mouse embryos at various developmental stages were subjected to immunostaining for DCC in A; integrin β1 subunit and F-actin in B; FAK and F-actin in C. DAPI was for nuclear staining. Figure S2, Green fluorescence protein (GFP) and red fluorescence protein (RFP) expression as an indicator of neogenin knock down and neogenin overexpression, respectively. After microinjection of neogenin-targeting shRNA vector that harbors conjugated GFP or co-injection of the neogenin cDNA vector and RFP vector into the 2-PN zygotes, the expression of GFP and RFP was visualized under a fluorescence microscope at the 2-cell and 4-cell stage, respectively. Left panel, phase-contrast images; middle and right panels, fluorescence images. Figure S3, Expression of neogenin in a blastocyst after microinjecting small hairpin RNA neogenin targeting vectors. (A) After microinjection of neogenin targeting shRNA vector into 2-PN zygotes, the expression level of neogenin in individual cells in a blastocyst was evaluated by immunostaining with anti-flag antibodies. In the left panel, scrambled neogenin shRNA vectors were microinjected (control). In the right panel, neogenin-targeting shRNA vectors were microinjected. DAPI, DAPI nucleus staining in blue; Anti-flag, visualization of the flag tag on neogenin in red; GFP, green fluorescence proteins in green; merged, superimposition of DAPI, anti-flag, and GFP. (B) Whole cell lysates of blastocysts were immunoblotted with anti-flag antibodies. GFP was used as a loading control. Scrambled shRNA, scrambled neogenin shRNA injection; Ng shRNA, neogenin-targeting shRNA injection. (C) The neogenin cDNA vectors or the neogenin-targeting shRNA vectors were microinjected into 2-PN zygotes and resulting blastocysts were subjected to immunostaining with anti-neo [file pone.0101989.s002.zip › Figure S3A and B.TIF]

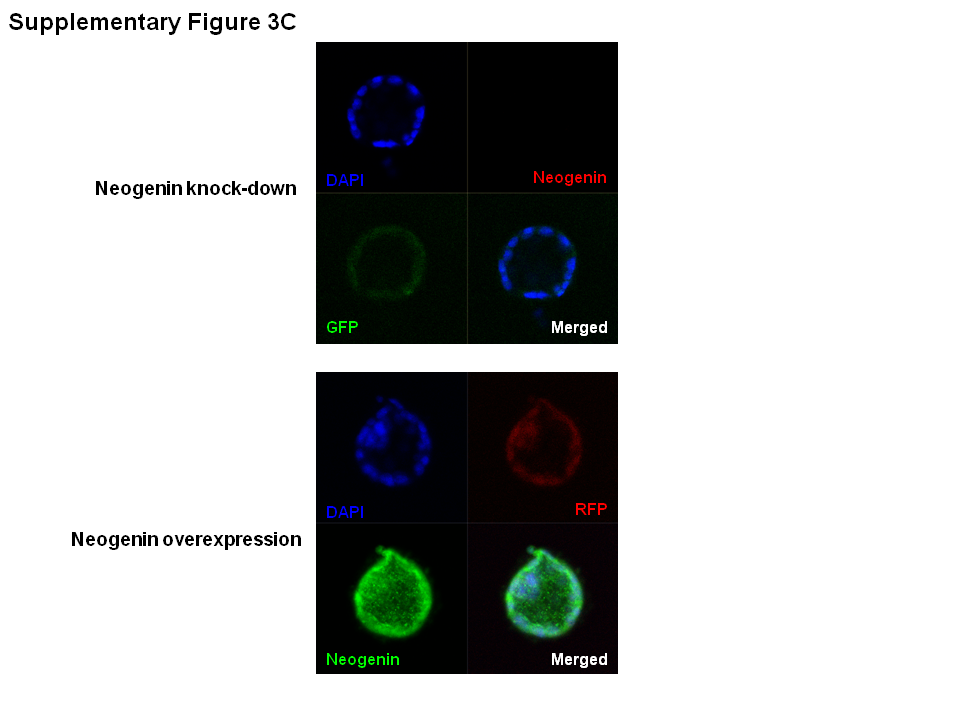

Supplement: File S1 — Supporting Figures. Figure S1, Expression profiles of focal adhesion kinase (FAK), F-action, integrin β1 subunit in mouse embryos viewed by immunostaining during early embryo development. Preimplantation mouse embryos at various developmental stages were subjected to immunostaining for DCC in A; integrin β1 subunit and F-actin in B; FAK and F-actin in C. DAPI was for nuclear staining. Figure S2, Green fluorescence protein (GFP) and red fluorescence protein (RFP) expression as an indicator of neogenin knock down and neogenin overexpression, respectively. After microinjection of neogenin-targeting shRNA vector that harbors conjugated GFP or co-injection of the neogenin cDNA vector and RFP vector into the 2-PN zygotes, the expression of GFP and RFP was visualized under a fluorescence microscope at the 2-cell and 4-cell stage, respectively. Left panel, phase-contrast images; middle and right panels, fluorescence images. Figure S3, Expression of neogenin in a blastocyst after microinjecting small hairpin RNA neogenin targeting vectors. (A) After microinjection of neogenin targeting shRNA vector into 2-PN zygotes, the expression level of neogenin in individual cells in a blastocyst was evaluated by immunostaining with anti-flag antibodies. In the left panel, scrambled neogenin shRNA vectors were microinjected (control). In the right panel, neogenin-targeting shRNA vectors were microinjected. DAPI, DAPI nucleus staining in blue; Anti-flag, visualization of the flag tag on neogenin in red; GFP, green fluorescence proteins in green; merged, superimposition of DAPI, anti-flag, and GFP. (B) Whole cell lysates of blastocysts were immunoblotted with anti-flag antibodies. GFP was used as a loading control. Scrambled shRNA, scrambled neogenin shRNA injection; Ng shRNA, neogenin-targeting shRNA injection. (C) The neogenin cDNA vectors or the neogenin-targeting shRNA vectors were microinjected into 2-PN zygotes and resulting blastocysts were subjected to immunostaining with anti-neo [file pone.0101989.s002.zip › Figure S3C.TIF]

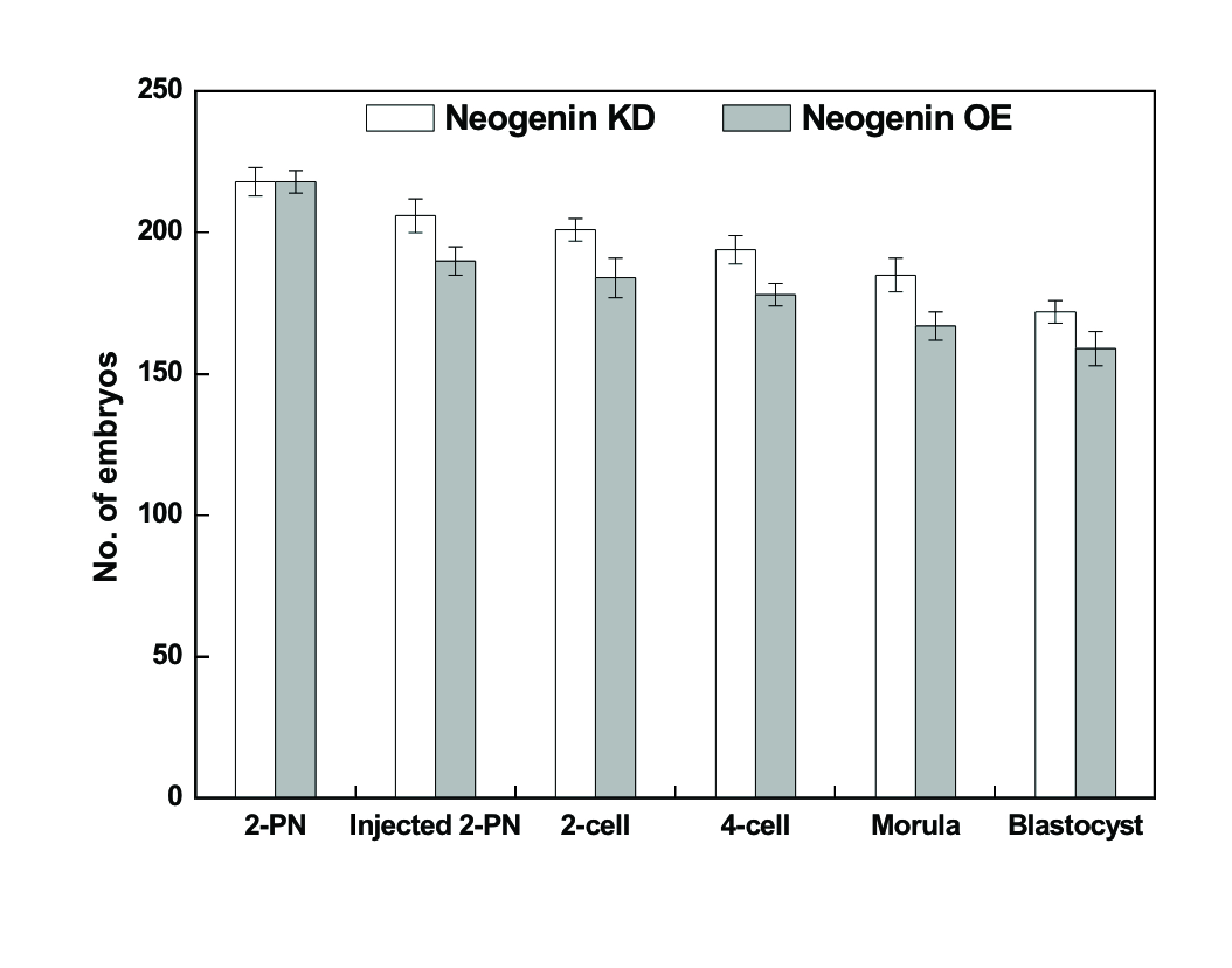

Supplement: File S1 — Supporting Figures. Figure S1, Expression profiles of focal adhesion kinase (FAK), F-action, integrin β1 subunit in mouse embryos viewed by immunostaining during early embryo development. Preimplantation mouse embryos at various developmental stages were subjected to immunostaining for DCC in A; integrin β1 subunit and F-actin in B; FAK and F-actin in C. DAPI was for nuclear staining. Figure S2, Green fluorescence protein (GFP) and red fluorescence protein (RFP) expression as an indicator of neogenin knock down and neogenin overexpression, respectively. After microinjection of neogenin-targeting shRNA vector that harbors conjugated GFP or co-injection of the neogenin cDNA vector and RFP vector into the 2-PN zygotes, the expression of GFP and RFP was visualized under a fluorescence microscope at the 2-cell and 4-cell stage, respectively. Left panel, phase-contrast images; middle and right panels, fluorescence images. Figure S3, Expression of neogenin in a blastocyst after microinjecting small hairpin RNA neogenin targeting vectors. (A) After microinjection of neogenin targeting shRNA vector into 2-PN zygotes, the expression level of neogenin in individual cells in a blastocyst was evaluated by immunostaining with anti-flag antibodies. In the left panel, scrambled neogenin shRNA vectors were microinjected (control). In the right panel, neogenin-targeting shRNA vectors were microinjected. DAPI, DAPI nucleus staining in blue; Anti-flag, visualization of the flag tag on neogenin in red; GFP, green fluorescence proteins in green; merged, superimposition of DAPI, anti-flag, and GFP. (B) Whole cell lysates of blastocysts were immunoblotted with anti-flag antibodies. GFP was used as a loading control. Scrambled shRNA, scrambled neogenin shRNA injection; Ng shRNA, neogenin-targeting shRNA injection. (C) The neogenin cDNA vectors or the neogenin-targeting shRNA vectors were microinjected into 2-PN zygotes and resulting blastocysts were subjected to immunostaining with anti-neo [file pone.0101989.s002.zip › Figure S4A.tif]

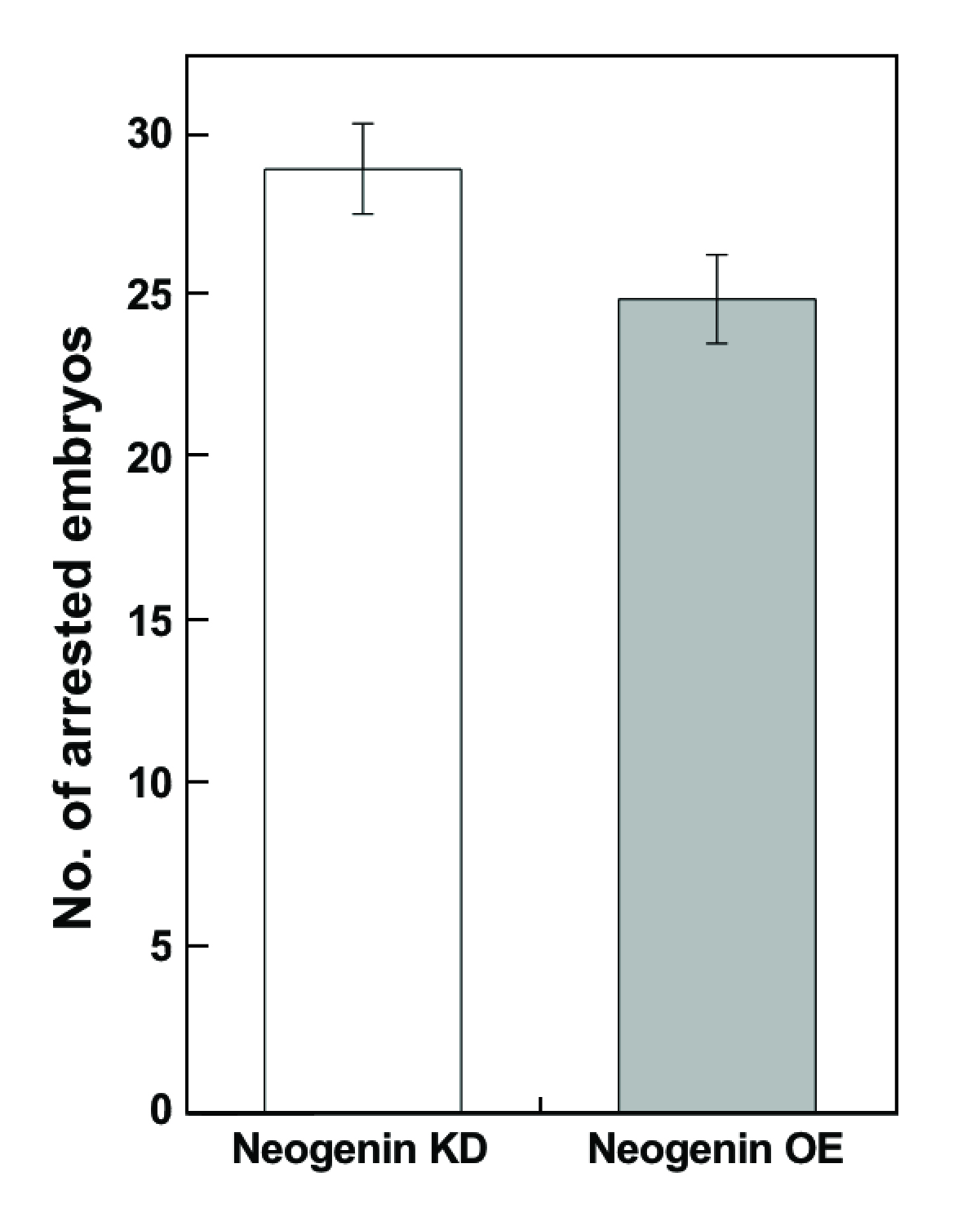

Supplement: File S1 — Supporting Figures. Figure S1, Expression profiles of focal adhesion kinase (FAK), F-action, integrin β1 subunit in mouse embryos viewed by immunostaining during early embryo development. Preimplantation mouse embryos at various developmental stages were subjected to immunostaining for DCC in A; integrin β1 subunit and F-actin in B; FAK and F-actin in C. DAPI was for nuclear staining. Figure S2, Green fluorescence protein (GFP) and red fluorescence protein (RFP) expression as an indicator of neogenin knock down and neogenin overexpression, respectively. After microinjection of neogenin-targeting shRNA vector that harbors conjugated GFP or co-injection of the neogenin cDNA vector and RFP vector into the 2-PN zygotes, the expression of GFP and RFP was visualized under a fluorescence microscope at the 2-cell and 4-cell stage, respectively. Left panel, phase-contrast images; middle and right panels, fluorescence images. Figure S3, Expression of neogenin in a blastocyst after microinjecting small hairpin RNA neogenin targeting vectors. (A) After microinjection of neogenin targeting shRNA vector into 2-PN zygotes, the expression level of neogenin in individual cells in a blastocyst was evaluated by immunostaining with anti-flag antibodies. In the left panel, scrambled neogenin shRNA vectors were microinjected (control). In the right panel, neogenin-targeting shRNA vectors were microinjected. DAPI, DAPI nucleus staining in blue; Anti-flag, visualization of the flag tag on neogenin in red; GFP, green fluorescence proteins in green; merged, superimposition of DAPI, anti-flag, and GFP. (B) Whole cell lysates of blastocysts were immunoblotted with anti-flag antibodies. GFP was used as a loading control. Scrambled shRNA, scrambled neogenin shRNA injection; Ng shRNA, neogenin-targeting shRNA injection. (C) The neogenin cDNA vectors or the neogenin-targeting shRNA vectors were microinjected into 2-PN zygotes and resulting blastocysts were subjected to immunostaining with anti-neo [file pone.0101989.s002.zip › Figure S4B.tif]

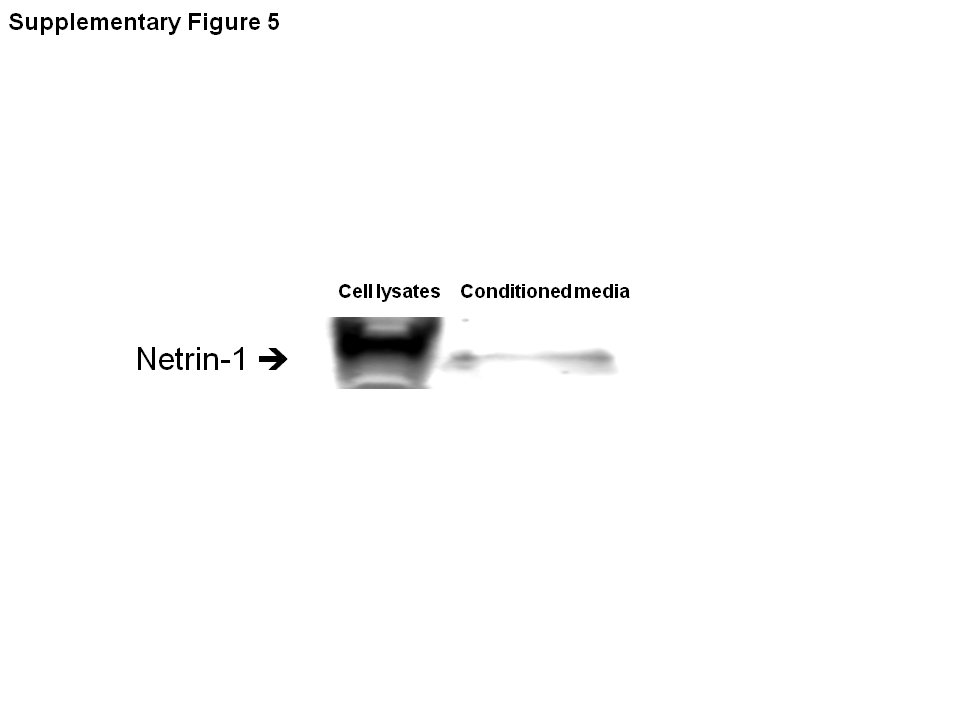

Supplement: File S1 — Supporting Figures. Figure S1, Expression profiles of focal adhesion kinase (FAK), F-action, integrin β1 subunit in mouse embryos viewed by immunostaining during early embryo development. Preimplantation mouse embryos at various developmental stages were subjected to immunostaining for DCC in A; integrin β1 subunit and F-actin in B; FAK and F-actin in C. DAPI was for nuclear staining. Figure S2, Green fluorescence protein (GFP) and red fluorescence protein (RFP) expression as an indicator of neogenin knock down and neogenin overexpression, respectively. After microinjection of neogenin-targeting shRNA vector that harbors conjugated GFP or co-injection of the neogenin cDNA vector and RFP vector into the 2-PN zygotes, the expression of GFP and RFP was visualized under a fluorescence microscope at the 2-cell and 4-cell stage, respectively. Left panel, phase-contrast images; middle and right panels, fluorescence images. Figure S3, Expression of neogenin in a blastocyst after microinjecting small hairpin RNA neogenin targeting vectors. (A) After microinjection of neogenin targeting shRNA vector into 2-PN zygotes, the expression level of neogenin in individual cells in a blastocyst was evaluated by immunostaining with anti-flag antibodies. In the left panel, scrambled neogenin shRNA vectors were microinjected (control). In the right panel, neogenin-targeting shRNA vectors were microinjected. DAPI, DAPI nucleus staining in blue; Anti-flag, visualization of the flag tag on neogenin in red; GFP, green fluorescence proteins in green; merged, superimposition of DAPI, anti-flag, and GFP. (B) Whole cell lysates of blastocysts were immunoblotted with anti-flag antibodies. GFP was used as a loading control. Scrambled shRNA, scrambled neogenin shRNA injection; Ng shRNA, neogenin-targeting shRNA injection. (C) The neogenin cDNA vectors or the neogenin-targeting shRNA vectors were microinjected into 2-PN zygotes and resulting blastocysts were subjected to immunostaining with anti-neo [file pone.0101989.s002.zip › Figure S5.TIF]

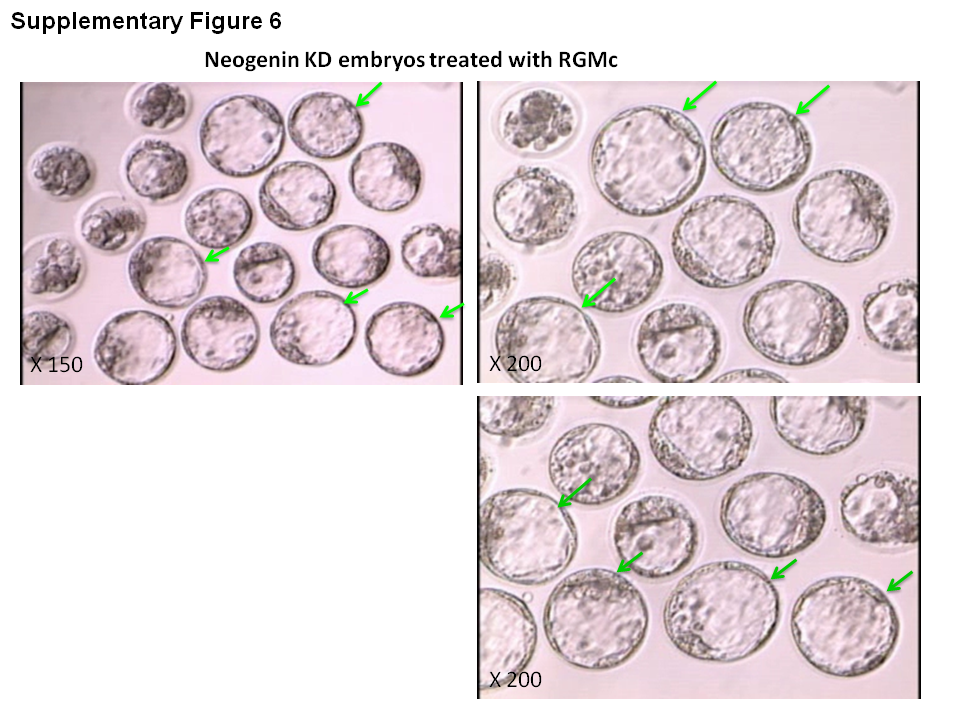

Supplement: File S1 — Supporting Figures. Figure S1, Expression profiles of focal adhesion kinase (FAK), F-action, integrin β1 subunit in mouse embryos viewed by immunostaining during early embryo development. Preimplantation mouse embryos at various developmental stages were subjected to immunostaining for DCC in A; integrin β1 subunit and F-actin in B; FAK and F-actin in C. DAPI was for nuclear staining. Figure S2, Green fluorescence protein (GFP) and red fluorescence protein (RFP) expression as an indicator of neogenin knock down and neogenin overexpression, respectively. After microinjection of neogenin-targeting shRNA vector that harbors conjugated GFP or co-injection of the neogenin cDNA vector and RFP vector into the 2-PN zygotes, the expression of GFP and RFP was visualized under a fluorescence microscope at the 2-cell and 4-cell stage, respectively. Left panel, phase-contrast images; middle and right panels, fluorescence images. Figure S3, Expression of neogenin in a blastocyst after microinjecting small hairpin RNA neogenin targeting vectors. (A) After microinjection of neogenin targeting shRNA vector into 2-PN zygotes, the expression level of neogenin in individual cells in a blastocyst was evaluated by immunostaining with anti-flag antibodies. In the left panel, scrambled neogenin shRNA vectors were microinjected (control). In the right panel, neogenin-targeting shRNA vectors were microinjected. DAPI, DAPI nucleus staining in blue; Anti-flag, visualization of the flag tag on neogenin in red; GFP, green fluorescence proteins in green; merged, superimposition of DAPI, anti-flag, and GFP. (B) Whole cell lysates of blastocysts were immunoblotted with anti-flag antibodies. GFP was used as a loading control. Scrambled shRNA, scrambled neogenin shRNA injection; Ng shRNA, neogenin-targeting shRNA injection. (C) The neogenin cDNA vectors or the neogenin-targeting shRNA vectors were microinjected into 2-PN zygotes and resulting blastocysts were subjected to immunostaining with anti-neo [file pone.0101989.s002.zip › Figure S6.TIF]

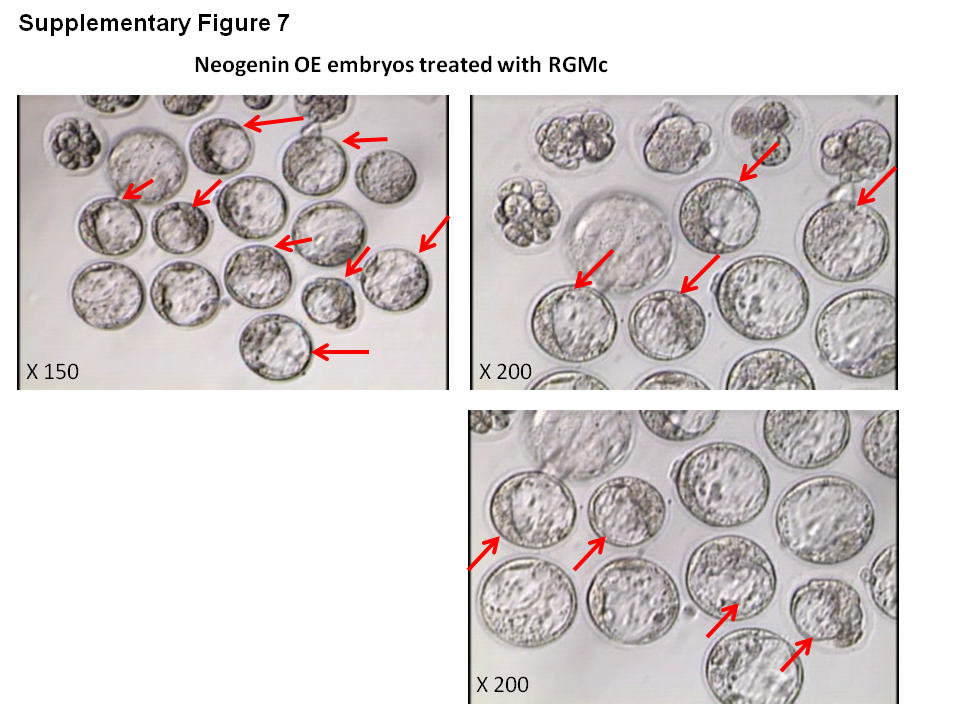

Supplement: File S1 — Supporting Figures. Figure S1, Expression profiles of focal adhesion kinase (FAK), F-action, integrin β1 subunit in mouse embryos viewed by immunostaining during early embryo development. Preimplantation mouse embryos at various developmental stages were subjected to immunostaining for DCC in A; integrin β1 subunit and F-actin in B; FAK and F-actin in C. DAPI was for nuclear staining. Figure S2, Green fluorescence protein (GFP) and red fluorescence protein (RFP) expression as an indicator of neogenin knock down and neogenin overexpression, respectively. After microinjection of neogenin-targeting shRNA vector that harbors conjugated GFP or co-injection of the neogenin cDNA vector and RFP vector into the 2-PN zygotes, the expression of GFP and RFP was visualized under a fluorescence microscope at the 2-cell and 4-cell stage, respectively. Left panel, phase-contrast images; middle and right panels, fluorescence images. Figure S3, Expression of neogenin in a blastocyst after microinjecting small hairpin RNA neogenin targeting vectors. (A) After microinjection of neogenin targeting shRNA vector into 2-PN zygotes, the expression level of neogenin in individual cells in a blastocyst was evaluated by immunostaining with anti-flag antibodies. In the left panel, scrambled neogenin shRNA vectors were microinjected (control). In the right panel, neogenin-targeting shRNA vectors were microinjected. DAPI, DAPI nucleus staining in blue; Anti-flag, visualization of the flag tag on neogenin in red; GFP, green fluorescence proteins in green; merged, superimposition of DAPI, anti-flag, and GFP. (B) Whole cell lysates of blastocysts were immunoblotted with anti-flag antibodies. GFP was used as a loading control. Scrambled shRNA, scrambled neogenin shRNA injection; Ng shRNA, neogenin-targeting shRNA injection. (C) The neogenin cDNA vectors or the neogenin-targeting shRNA vectors were microinjected into 2-PN zygotes and resulting blastocysts were subjected to immunostaining with anti-neo [file pone.0101989.s002.zip › Figure S7.TIF]
